# Inflatable penile prosthesis implantation performed by residents after a structured training pathway

**DOI:** 10.1111/bju.70028

**Published:** 2025-10-10

**Authors:** Liliana Guadagni, Marco Falcone

**Affiliations:** ^1^ Urology Clinic, Department of Surgical and Biomedical Sciences, Santa Maria della Misericordia Hospital University of Perugia Perugia Italy; ^2^ Urology Clinic, A.O.U. ‘Città della Salute e della Scienza’, Molinette Hospital University of Turin Turin Italy; ^3^ Neurourology Clinic, A.O.U. ‘Città della Salute e della Scienza’ Unità Spinale Unipolare Turin Italy; ^4^ Department of Urology Biruni University School of Medicine İstanbul Turkey

**Keywords:** penile prosthesis implant, resident training, erectile dysfunction, inflatable penile prosthesis, cadaver laboratory, surgical outcomes, prosthetic training programme

**Abbreviations**
3D: three‐dimensional
lab: laboratory
PPI: penile prosthesis implantation

Erectile dysfunction is a condition affecting up to 60% of men aged >40 years. It can be caused by various factors, including organic (vasculogenic, neurogenic, anatomical, or hormonal), drug‐induced, psychological, or a combination of them [1].

Patient preference, lack of response to behavioural interventions and pharmaceutical treatments are underlying conditions leading to considerations of a penile prosthesis implantation (PPI), either semi‐rigid or inflatable three‐piece [1].

Implants are burdened with complications, such as infection and mechanical failure. Currently, complication rates reported in literature are <5% after 5 years of follow‐up [1].

To ensure minimal complications and better functional outcomes, the implant should be performed by an experienced prosthetic urologist [1]. Despite this fact, only 15% of urology training programmes in the United States have a dedicated prosthetic urologist teaching programme [2]. To increase the number of trained prosthetic specialists, it would be necessary to build a globally shared training programme dedicated to residents in urology. Following these leads, recent evidence highlighted how a cadaver laboratory (lab) training programme may lead to a significant improvement in procedural knowledge and understanding the differences between the types of penile prosthesis [3]. We strongly believe that a properly structured training programme could positively affect the overall quality of prosthetic surgeries worldwide.

With the lack of an actual standardised PPI training programme, our aim was to present a proposal of a resident educational programme and to evaluate its preliminary results, comparing the surgical outcomes of PPI performed by the leading consultant or a trained resident. A further step was providing insightful suggestions for the development of a prosthetic training programme to be reproduced worldwide.

The study was performed in accordance with the Declaration of Helsinki and in compliance with European Union Regulation 2016/679 (General Data Protection Regulation). Data were gathered retrospectively (protocol number: 0041021).

For a proper setting, a high‐volume tertiary care facility allowing the residents to deal with a large number of PPIs surgery is deemed to be necessary. In our centre, currently around 60 PPI are performed every year.

From January 2022 to March 2025, our institution offered a PPI training programme involving senior residents in their fourth and fifth years of their residency programme. The director of the training programme was a senior consultant with broad experience in reconstructive urology, andrology, and prosthetic urology (M.F.).

The programme followed five fundamental steps consisting in sequential levels of training: development of soft skills based on 10 h of lessons focusing on penile prosthetic surgery (history of penile prosthesis, technical features of penile prosthesis, pre‐, peri‐ and postoperative care of patients undergoing PPI, step‐by‐step surgical procedure, management of complications, and difficult cases of PPI, such as fibrosis, penile curvature, revisions); hands on training assisting in the operative room with the leading consultant (M.F.) in at least 20 cases of PPI; cadaver lab training with *ex vivo* PPI; PPI *in vivo* with leading consultant assistance (at least 10 cases required); the last step is solo PPI under leading consultant surveillance, recording operative time and the number of mistakes for every step of the surgery.

To date, three residents (Residents A, B, and C) adhered to our programme along the fourth and fifth years of their residency. The following data of PPI procedures were retrospectively collected: operative time, hospital stay, follow‐up, and complications following the Clavien–Dindo classification [4]. Patients undergoing PPI performed by a resident alone were considered as cases, whereas patients undergoing PPI performed by the leading consultant (M.F.) assisted by a resident in training were considered as the control. All patients had an inflatable penile prosthesis implanted through penoscrotal access to ensure a standardised procedure.

The exclusion criteria for patients were previous penile surgeries, radiotherapy, current therapy with anticoagulants, penile curvature, or important comorbidities (Charlson Comorbidity Index ≥1).

We examined the difference between the consultant cases and resident cases in patient factors (age, body mass index) and surgical outcomes (operative time and length of hospital stay) plus follow‐up time with the Mann–Whitney test. Categorical outcomes (diabetes, smoking status, and complications) were compared with the Fisher's exact test. Statistical analyses were performed using Jamovi software [5].

From January 2022 to February 2025, 200 consecutive patients underwent PPI in our institution. Among them, 50 patients (median [interquartile range] age 66 [59–70] years) adhered to the inclusion criteria and were therefore enrolled in the present study. In all, 31 patients were included in the case group (residents), whereas the rest were allocated to the control group (consultant).

The surgical outcomes of PPI are summarised in Table 1.

**Table 1:** Outcomes of the surgeries performed by the residents and consultant.

Variable	Leading consultant (*n* = 19)	Residents (*n* = 31)	*P*
Patient age, years, median (IQR)	64 (57–69)	66 (61–72)	0.170
BMI, kg/m^2^, median (IQR)	26 (24–26)	24 (24–26)	0.094
Current or former smoker, *n* (%)	7 (36.9)	9 (29.0)	0.419
Type II diabetes, *n* (%)	5 (26.3)	7 (22.7)	0.770
Type of penile prosthesis: Titan® (Coloplast, Minneapolis, MN, USA); AMS® (Boston Scientific, Marlborough, MA, USA), *n* (%)	11 (57.9); 8 (42.1)	15 (48.4); 16 (51.6)	0.523
Operative time, min, median (IQR)	90 (77–102)	80 (66–97)	0.199
Hospital stay, days, median (IQR)	2 (2–2)	2 (2–2)	0.560
Complications classified by Clavien–Dindo Grade, *n* (%)	1 (5.3)	2 (6.5)	>0.9
Follow‐up, weeks, median (IQR)	95 (77–105)	67 (36–78)	<0.001

IQR, interquartile range, Q1–Q3; BMI, body mass index.

The longest hospital stay was 3 days and occurred for 8% of patients (two in each group).

Three cases of complications classified using the Clavien–Dindo scale were detected [4]. A single patient in the resident group experienced an episode of a transient fever a week after PPI, and it was treated by oral antibiotic therapy (Grade I). Two cases required a surgical revision (Grade III): a substitution of PPI for mechanical failure and a case of glandulopexy for floppy glans syndrome (in the consultant and resident groups, respectively).

The fact that many young surgeons receive minimal hands‐on surgical training is a well‐known and widespread limitation in worldwide surgical education. The preliminary results of our structured PPI training programme highlighted satisfactory results, reporting comparable surgical outcomes when comparing surgeries performed by a leading consultant or a fully trained resident. The slight difference in median operative time between the groups is likely due to the training process itself, as the consultant showed each step slowly so that the residents could fully understand the procedure. The limited incidence of complications detected in our series was in line with what has been reported in the current literature [6]. In addition, we all may agree in considering mechanical failure as a PPI complication not related to surgeon experience or skills. In the current literature, few authors discussed training programmes of residents in Urology. A procedurally focused cadaver course that covered a wide range of fundamental surgical procedures determined a significant improvement of self‐reported operating confidence and competence [3, 7]. A penile three‐dimensional (3D)‐printed model composed of a synthetic hydrogel tissue mimicking the biomechanical properties of human tissue was designed in 2020 [8]. In the near future, we aim to integrate PPI in 3D‐printed models as a preceding step to the cadaver lab.

Our study has several limitations. First, the number of residents participating in our prosthetic training was limited, but we hope to involve more participants in the future. The retrospective nature of the study, as well as its monocentric design, represent limitations in regard to the quality of the data. Upgrading our study to a multicentric setting would increase the reliability of the data as well as create a panel of experts in prosthetic urology that could further develop the training programme.

Our programme is showing encouraging results on training residents in PPI. We strongly believe that our community should invest in the development of a shared and structured penile prosthesis training programme to improve the skills of future generations of urological surgeons.

## Disclosure of Interests

The authors declare no conflict of interest.

## References

[1] EAU Guidelines . Edn. presented at the EAU Annual Congress, Madrid 2025. ISBN 978‐94‐92671‐29‐5

[2] Kovac JR . Centers of excellence for penile prosthetics are a novel concept that will likely prove difficult to implement. In: Translational Andrology and Urology , editor. Translational Andrology and Urology, Vol. 6. Hong Kong: AME Publishing Company, 2017: S898–S899 29239399 10.21037/tau.2017.11.21 PMC5715184

[3] Lentz AC , Rodríguez D , Davis LG et al. Simulation training in penile implant surgery: assessment of surgical confidence and knowledge with cadaveric laboratory training. Sex Med 2018; 6: 332–338 30454614 10.1016/j.esxm.2018.09.001 PMC6302135

[4] Naumann DN , Vincent LE , Pearson N et al. An adapted Clavien‐Dindo scoring system in trauma as a clinically meaningful nonmortality endpoint. J Trauma Acute Care Surg 2017; 83: 241–248 28731937 10.1097/TA.0000000000001517 PMC5527750

[5] The Jamovi Project . Jamovi. (Version 2.6) [Computer Software]. 2024. Available at: https://www.jamovi.org

[6] Miller LE , Khera M , Bhattacharyya S , Patel M , Nitschelm K , Burnett AL . Long‐term survival rates of inflatable penile prostheses: systematic review and meta‐analysis. Urology 2022; 166: 6–10 35421510 10.1016/j.urology.2022.03.026

[7] Sharma G , Aycart MA , Najjar PA et al. A cadaveric procedural anatomy course enhances operative competence. J Surg Res 2016; 201(1): 22–28 26850180 10.1016/j.jss.2015.09.037

[8] van Renterghem K , Ghazi A . 3D pelvic cadaver model: a novel approach to surgical training for penile implant surgery. Int J Impot Res 2020; 32: 261–263 31649337 10.1038/s41443-019-0211-2 PMC8076011

